# Bioactive Compounds from the Roots of *Asiasarum heterotropoides*

**DOI:** 10.3390/molecules19010122

**Published:** 2013-12-23

**Authors:** Jun Lee, You Jin Lee, Se-Mi Oh, Jin-Mu Yi, No Soo Kim, Ok-Sun Bang

**Affiliations:** KM-Based Herbal Drug Development Group, Herbal Medicine Research Division, Korea Institute of Oriental Medicine, Daejeon 305-811, Korea; E-Mails: junlee@kiom.re.kr (J.L.); ojung8384@kiom.re.kr (Y.J.L.); sem5630@kiom.re.kr (S.-M.O.); jmyi@kiom.re.kr (J.-M.Y.); nosookim@kiom.re.kr (N.S.K.)

**Keywords:** *Asiasarum heterotropoides*, aristolochiaceae, tetrahydrofuran lignan, cytotoxicity, nitric oxide, FOXP3, HIF-1α

## Abstract

A new tetrahydrofuran lignan, (7*S*,8*R*,7'*S*,8'*S*)-3-methoxy-3',4'-methylenedioxy-7,9'-epoxylignane-4,7',9-triol (**1**), and 21 known compounds **2**–**22** were isolated from the roots of *Asiasarum heterotropoides* by chromatographic separation methods. The structures of all compounds **1**–**22** were elucidated by spectroscopic analysis including 1D- and 2D-NMR. Fourteen of these compounds (**1**–**3**, **7**, **10**, **12**–**17**, **19**, **21**, and **22**) were isolated from this species in this study for the first time. All of the isolates were evaluated for their anticancer activities using *in vitro* assays. Among the 22 tested compounds, two (compounds **5** and **7**) induced the downregulation of NO production, FOXP3 expression, and HIF-1α transcriptional activity.

## 1. Introduction

*Asiasarum heterotropoides* F. Maekawa var. *mandshuricum* F*.* Maekawa (Aristolochiaceae), a deciduous perennial herb, is widely distributed in East Asia, Europe, and North America [1]. The dried roots of this herb and those of *Asiasarum sieboldii* F. Maekawa are known as Asiasari radix and have been used for the treatment of coughs, headaches, toothaches, rheumatism, aphthous stomatitis, local anesthesia, and inflammatory diseases in Traditional Chinese Medicine (TCM) [2,3,4]. Asiasari radix extracts have been shown to have hyperalgesia inhibitory, antitussive, immunoglobulin E inhibitory, memory enhancing, hair growth promoting, melanogenesis inhibitory, and anticancer effects [4,5,6,7,8,9].

Some phytochemical and pharmacological studies of *A. heterotropoides* have reported several types of secondary metabolites, including essential oils, monoterpenes, lignans, alkaloids, and phenyl propanoids, that display antimicrobial, anti-tumor, anti-inflammatory, and larvicidal activities [10,11,12,13,14]. Despite their medicinal importance and availability, knowledge of the chemical constituents and biological activities of *A. heterotropoides* remains insufficient to evaluate their pharmacological effects.

In the course of our research to discover anticancer agents from medicinal herbs, the chromatographic separation of an 80% EtOH extract from the roots of *A. heterotropoides* resulted in the isolation and identification of a new tetrahydrofuran lignan (**1**) and 21 known compounds **2**–**22**. The structures of all compounds **1**–**22** were elucidated by spectroscopic analysis, including NMR, and through comparison of the data with published values. All of the compounds **1**–**22** were evaluated for cytotoxic activity against human cancer cell lines and for inhibitory effects on nitric oxide production, FOXP3 promoter activation, and HIF-1α transcriptional activity. Here, we report the isolation, structural elucidation, and biological activities of these compounds from the roots of *A. heterotropoides*.

## 2. Results and Discussion

**Figure 1:**
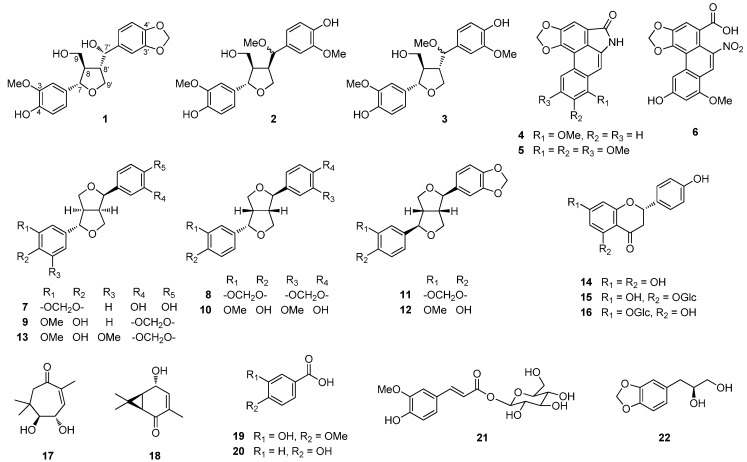
Structures of compounds **1**–**22** from the roots of *A. heterotropoides*.

Phytochemical investigation of the roots of *A. heterotropoides* led to the isolation of a new tetrahydrofuran lignan (**1**) and 21 known compounds **2**–**22** including two tetrahydrofuran lignans **2** and **3**, two aristolactams **4** and **5**, the aristolochic acid derivative **6**, seven tetrahydrofurofuran lignans **7**–**12**, three flavanones **14**–**16**, two monoterpenes **17** and **18**, and four phenolics **19**–**22** by chromatographic separation methods. The structures of all isolates **1**–**22** were elucidated through analysis of their spectroscopic data, including 1D- and 2D-NMR, and comparison of these data with the reported values (Figure 1).

**Figure 2:**
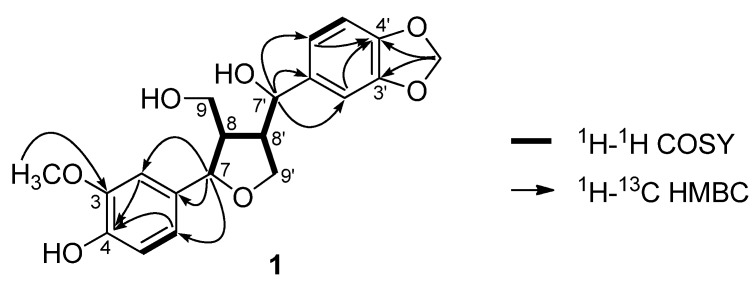
Important COSY and HMBC correlations of **1**.

Compound **1** was obtained as an amorphous solid and found to have the molecular formula C_20_H_22_O_7_, derived from the HRESIMS peak at *m/z* 397.1263 [M+Na]^+^ (calcd. for C_20_H_22_O_7_Na, 397.1263), suggesting 10 degrees of unsaturation. The UV spectrum of **1** showed characteristic absorption maxima at 227 and 283 nm. The ^1^H-NMR spectrum of **1** showed signals for the six protons of two 1,3,4-trisubstituted phenyl groups [*δ*_H_ 6.86 (3H, m, overlapping), 6.79 (1H, m), 6.77 (1H, m), and 6.75 (1H, d, *J* = 8.0 Hz)], a methylenedioxy group [*δ*_H_ 5.96 (2H, s)], and a methoxy group [*δ*_H_ 3.89 (3H, s)], indicating the presence of a piperonyl group and a vanillyl group. The presence of two oxygenated benzylic methines, two oxygenated methylenes, and two aliphatic methines were also confirmed from the ^1^H-, ^13^C-, and DEPT NMR spectra. The 2D-NMR data suggested an 8,8'-linked tetrahydrofuran skeleton, which was confirmed by correlations between H-8 and H-7/H-9/H-8' and between H-8' and H-7'/H-9'/H-8 in the ^1^H-^1^H COSY spectrum (Figure 2). The HMBC cross peaks of H-7 [*δ* 4.34 (1H, d, *J* = 9.0 Hz)] with C-1/C-2/C-6 and of H-7' [*δ* 4.41 (1H, d, *J* = 9.5 Hz)] with C-1'/C-2'/C-6' demonstrated that the vanillyl and piperonyl groups are linked to C-7 and C-7', respectively. The position of the methoxy group was determined from its correlation with C-3 in the HMBC spectrum. Furthermore, the positions of the oxygenated quaternary carbons (C-3/C-4/C-3'/C-4') in the phenyl rings were also determined by the HMBC cross peaks (C-4/H-2,H-6, C-4'/H-2',H-6') (Figure 2).

**Figure 3:**
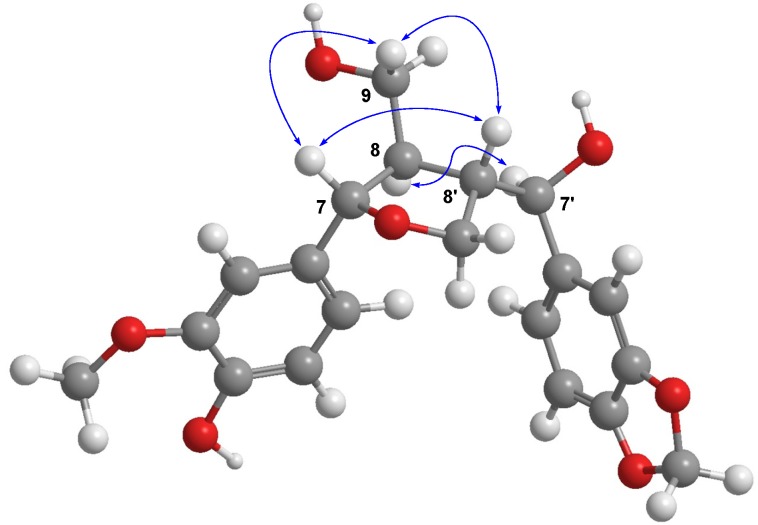
MM2 energy-minimized 3D structure using ChemBio3D Ultra (ver. 12.0) and key NOESY correlations of **1**.

Therefore, compound **1** was found to be a new tetrahydrofuran lignan, 3-methoxy-3',4'-methylenedioxy-7,9'-epoxylignane-4,7',9-triol. The relative configurations between H-7, H-8, and H-8' were determined to be the all *trans* orientation by the NOE cross peaks of H-7/H-9, H-7/H-8', H-8'/H-9, and H-8/H-7' in the NOESY spectrum (Figure 3).

The absolute configurations at C-7 and C-7' were determined by comparison of the optical rotation value and circular dichroism (CD) data with those of analogous compounds. The CD spectrum of **1** (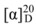 +39.2, MeOH) showed positive signals at 245 and 293 nm [15,16,17]. Therefore, compound **1** was determined to be (7*S*,8*R*,7'*S*,8'*S*)-3-methoxy-3',4'-methylenedioxy-7,9'-epoxylignane-4,7',9-triol.

The 21 known compounds **2**–**22** were identified as (+)-7'-methoxylariciresinol (**2**) [18],(–)-tanegool-7'-methyl ether (**3**) [18], aristolactam I (**4**) [19], 7-methoxyaristololactam IV (**5**) [20,21], aristolochic acid IVa (**6**) [22], (7*α*,7'*β*,8*α*,8'*α*)-3,4-methylenedioxy-3',4'-dihydroxy-7,9':7',9-diepoxy-lignane (**7**) [23], (–)-asarinin (**8**) [24], (+)-xanthoxylol (**9**) [25], (–)-epipinoresinol (**10**) [25],(–)-sesamin (**11**) [26], (–)-piperitol (**12**) [27,28], (1*R*,2*S*,5*R*,6*R*)-5'-*O*-methylpluviatilol (**13**) [29], (2*S*)-naringenin (**14**) [30], (2*S*)-naringenin-5-*O*-*β*-d-glucopyranoside (**15**) [31], (2*S*)-naringenin-7-*O*-*β*-d-glucopyranoside (**16**) [32], (+)-asarinol D (**17**) [33], (±)-asarinol A (**18**) [34], isovanillic acid (**19**) [35], 4-hydroxybenzoic acid (**20**) [36], *trans*-*p*-feruloyl-*β*-d-glucopyranoside (**21**) [37], and (*R*)-5-(2,3-dihydroxypropyl)-1,3-benzodioxole (**22**) [38]. Thirteen of these compounds (**2**, **3**, **7**, **10**, **12**–**17**, **19**, **21**, and **22**) were isolated from *A. heterotropoides* for the first time in this study.

**Figure 4:**
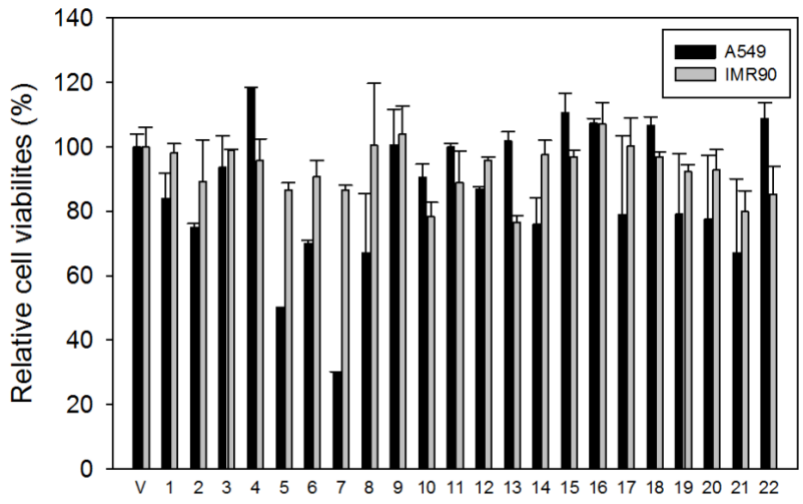
Effects of compounds **1**–**22** on cell viability. A549 lung cancer cells and IMR90 normal lung cells were exposed to 20 μM compound (**1**–**22**) or vehicle (V, DMSO). After 48 h, the cell viability was quantified, and the relative viabilities of each compound were determined by comparing with vehicle treatment. The data are presented as the means ± S.D. of three independent experiments.

Our previous study showed that ethanol extracts of Asiasari radix have anticancer effects on human non-small cell lung carcinoma (NSCLC) cells [9]; therefore, we examined the biological activities of the 22 compounds related with anticancer effects. First, the cytotoxic effects of the isolated compounds were investigated using A549 human NSCLC cells and IMR90 human normal lung fibroblast cells. The cells were exposed to the test compounds (20 μM) or vehicle (DMSO) for 48 h and the cell viabilities were determined based on the mitochondrial dehydrogenase activities (Ez-Cytox). Remarkably, compounds **5** and **7** inhibited the growth of A549 lung cancer cells at 20 μM, as shown in Figure 4. The viability of A549 cells was reduced to 50% and 30% by compounds **5** and **7**, respectively, when compared with the vehicle treatment. Notably, compounds **5** and **7** only marginally affected the growth of IMR90 normal lung cells, and their viabilities were maintained at over 85% using the same compound concentration (20 μM). Therefore, compounds **5** and **7** selectively killed A549 lung cancer cells without affecting normal cells.

**Figure 5:**
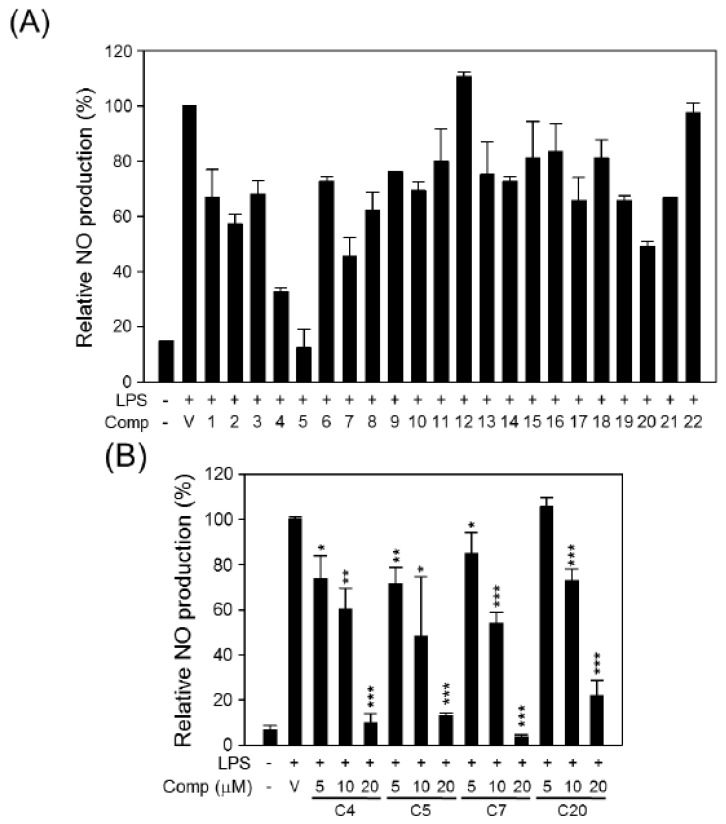
Effects of compounds on NO production. (**A**) Raw264.7 cells were treated with a combination of 1 μg/mL LPS and 20 μM compound or vehicle (V, DMSO) as a control. (**B**) Raw264.7 cells were treated with combination of 1 μg/mL LPS and the indicated concentrations of selected compounds. Extracellular NO was quantified and relative NO production was determined by comparing with LPS (+)/vehicle (V) treatment group. The data are presented as the means ± S.D. of three independent experiments. Differences between each treatment groups and LPS control group (LPS (+)/vehicle (V)) were compared and statistical significances are denoted as * *p* < 0.05, ** *p* < 0.01 or *** *p* < 0.001.

Nitric oxide (NO) synthesized from L-arginine by a group of nitric oxide synthases has been shown to promote tumor growth by regulating the expression of genes involved in cell mobility, invasion, and angiogenesis [39,40,41]. Chronic and substantial exposure to NO was also reported to contribute to cancer development [42,43]. In addition, the application of a highly selective NOS inhibitor, *N*^G^-monomethyl-l-arginine, monoacetate and the consequent reduction of the NO concentration was shown to sensitize radio-resistant NSCLC cells to radiotherapy [44]. Therefore, the inhibition of up-regulated NO production should be considered an anticancer strategy. In the present study, we used LPS-mediated NO production from Raw264.7 murine macrophage cells as an *in vitro* screening system to investigate the potential of the isolated compounds to inhibit NO production. As shown in Figure 5, four compounds (**4**, **5**, **7**, and **20**) reduced the production of NO by more than 50% by Raw264.7 cells treated with 1 μg/mL LPS when compared with vehicle treatment (Figure 5A), and their inhibitory effects were observed to be in dose-dependent (Figure 5B).

**Figure 6:**
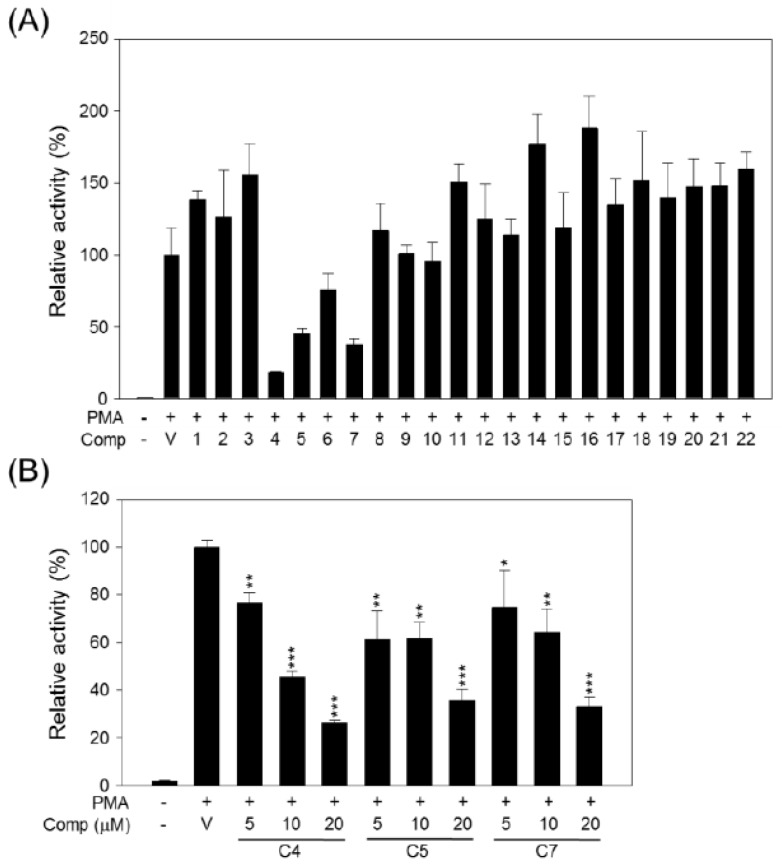
Effects of compounds on FOXP3 promoter activity. (**A**) FOXP3-Luc#3 cells were exposed to combinations of PMA (5 ng/mL) and each compound (20 μM) or vehicle as a control (V, DMSO); and (**B**) FOXP3-Luc#3 cells were exposed to increasing concentrations of each selected compound (compounds **4** (C4), **5** (C5), or **7** (C7)). After 24 h incubation, luciferase activity in the whole cell lysate (WCL) was quantified, and the relative activity was determined by comparing with the PMA (+)/vehicle (V) treatment group. The data are presented as the means ± S.D. of three independent experiments. Differences between each treatment groups and the PMA control group (PMA (+)/vehicle (V)) were compared and statistical significances are denoted as * *p* <0.05, ** *p* < 0.01 or *** *p* < 0.001.

As a member of the forkhead box (FOX) family protein, FOXP3 is a master regulator of regulatory T cells (T_reg_) and functions in the maintenance of immune tolerance [45]. Abnormally high expression of FOXP3 was observed in various types of tumors [46,47,48,49] and was known to affect the sensitivity of cancer cells to anticancer drugs [50] and radiotherapy [51]. Therefore, the down-regulation of FOXP3 is one antitumor strategy. In this study, we investigated the effects of isolated compounds on FOXP3 expression using the FOXP3 promoter-luciferase reporter system, FOXP3-Luc#3 stable cells. The expression of luciferase in FOXP3-Luc#3 cells was derived by phorbol 12-myristate 13-acetate (PMA) treatment. As shown in Figure 6A, compounds **4**, **5**, and **7** significantly inhibited the PMA-mediated FOXP3 promoter activation by 82%, 55%, and 63%, respectively, compared with vehicle treatment. In addition compounds **4**, **5**, and **7** could inhibit FOXP3 promoter activity in a dose-dependent manner (Figure 6B). The vehicle, as the control (V, 0.1% DMSO), did not affect PMA-induced FOXP3 promoter activity.

**Figure 7:**
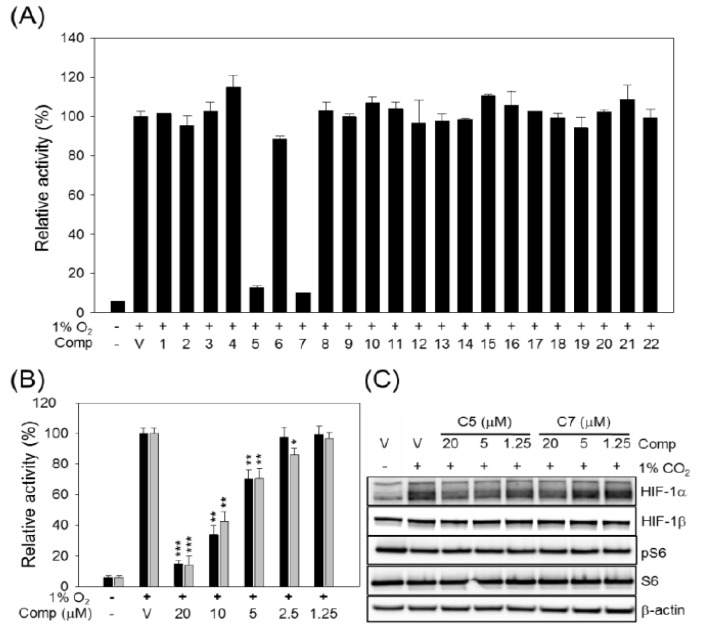
Effects of compounds on HIF-1α transcriptional activity. (**A**) NIH3T3/HIF-luc cells were pretreated with 20 μM of compound or vehicle (V, DMSO) for 2 h and then transferred to a hypoxic chamber (1% O_2_, 5% CO_2_, N_2_ balanced). After 24 h, the luciferase activity in the WCL was determined; (**B**) Dose dependent inhibition of HIF-1α transcriptional activity was observed with compounds **5** (black bar) and **7** (gray bar). The relative activities were determined by comparing with the hypoxia control (1% O_2_ (+)/vehicle (V)). The data are presented as the means ± S.D. of three independent experiments. The differences between each treatment group and the hypoxia control group (1% O_2_ (+)/vehicle (V)) were compared and statistical significance is denoted as * *p* < 0.05, ** *p* < 0.01 or*** *p* < 0.001 and (**C**) Compounds **5** and **7** inhibited hypoxic gas-induced HIF-1α expression in A549 cells in a dose-dependent manner. β-actin was applied as a loading control.

As a key regulator during tumor development, hypoxia inducible factor-1 (HIF-1), in particular HIF-1α is a promising target for anticancer drug development. To investigate whether the isolated compounds could modulate the transcriptional activity of HIF-1α under hypoxic stress, we performed a luciferase reporter assay using a NIH3T3/HIF-luc stable cell line carrying hypoxia response element (HRE) sequences. To introduce hypoxic stress, the cells were transferred into a hypoxic chamber containing 1% O_2_ and maintained for 24 h. Cells were pretreated with compound or vehicle 2 h before exposure to hypoxic gas. At the end of hypoxic stress, WCL was prepared and applied to the luciferase activity assay. As shown in Figure 7A, hypoxic stress increased luciferase activity up to 20-folds, compared with the normoxic condition (20% O_2_). Among the twenty-two tested compounds, two (compounds **5** and **7**) successfully inhibited HIF-1α transcriptional activity. Compounds **5** and **7** significantly inhibited hypoxic gas-induced luciferase activity by 92.5% and 94.6%, respectively, compared with the hypoxia control (1% O_2_ (+), vehicle treatment). As expected, both compounds could dramatically inhibit HIF-1α transcriptional activity in a dose-dependent manner (Figure 7B). Their effective doses at 50% inhibition (ED_50_) were 7.6 and 8.1 μM, respectively.

To elucidate the mechanism of the decrease in HIF-1α transcriptional activity induced by compounds **5** and **7**, we first investigated changes in the intracellular levels of HIF-1α in A549 human lung cancer cells treated with a combination of 1% O_2_ exposure and compound pretreatment. Increasing concentrations of compounds **5** and **7** were applied to A549 cells 2 h before exposure to hypoxic gas. After 20 h, the total protein was extracted, and expression of intracellular HIF-1α was visualized by western blotting. As shown in Figure 7C, hypoxic gas-induced HIF-1α expression was reduced by compounds **5** (C5) and **7** (C7) in a dose-dependent manner. However, HIF-1β, which is constitutively expressed, was not affected by C5 and C7. Furthermore, we investigated the PI3K/Akt/mTOR signaling, a HIF-1α synthetic pathway, by observing phosphorylation of the S6 ribosomal protein, which is the substrate of the PI3K/Akt/mTOR downstream kinase, p70 S6 kinase. As shown in Figure 7C, neither C5 nor C7 affected the level of phosphorylated S6 protein. Taken together, compounds **5** and **7** can inhibit HIF-1α transcriptional activity (Figure 7B) by decreasing intracellular levels of HIF-1α by regulating its stability.

Previous pharmacological studies have reported that compounds **5** and **7** showed cytotoxic activity against only a human proximal tubular epithelial cell line (HK-2) [52,53]. To the best of our knowledge, this is the first biological report of the inhibitory effects of compounds **4**, **5**, and **7** on NO, FOXP3, and HIF-1α.

Resveratrol can protect RAW264.7 cells from LPS-mediated inflammatory insults by inhibition of proinflammatory mediators including NO [54]. Al Dhaheri *et al.* demonstrated that ethanolic extract of *Origanum majorana* exerts its antitumor and antimetastatic potential in MDA-MB-231 breast cancer cells through down-regulation of NO production [40]. Aizman *et al.* demonstrated that farnesylthiosalycilic acid can down-regulate FoxP3 protein by Ras inhibition leading to induction of anti-tumor cytotoxic T-cell reactivity in glioma cells [55]. A bunch of previous reports showed that single compounds which can down-regulate HIF-1 signaling exert anticancer activities in diverse *in vitro* and *in vivo* systems [56,57,58,59,60,61].

## 3. Experimental

### 3.1. General

Optical rotations were obtained using a P-2000 polarimeter (Jasco, Tokyo, Japan). The UV spectra were measured on a CARY 400 Bio spectrophotometer (Varian, Palo Alto, CA, USA). The CD spectra were measured on a J-710 spectropolarimeter (Jasco). The HRESIMS were obtained using a hybrid quadrupole orthogonal time-of-flight (Q-TOF) mass spectrometer (SYNAPT G2, Waters, MS Technologies, Manchester, UK) coupled with an electrospray ionization (ESI) source. The NMR experiments were conducted on an Advance 500 FT-NMR (Bruker, Rheinstetten, Germany), with tetramethylsilane (TMS) as an internal standard. Thin layer chromatography (TLC) analysis was performed on Silica gel 60 F_254_ (Merck, Darmstadt, Germany) and RP-18 F_254S_ (Merck) plates. Silica gel (230–400 mesh, Merck), reversed-phase silica gel (YMC, ODS-A, 12 nm, S-150 μm, Kyoto, Japan), Sephadex LH-20 (Sigma-Aldrich, St. Louis, MO, USA), and Cosmosil 140C_18_-OPN (Nacalai Tesque, Kyoto, Japan) were used for chromatographic separation. Flash chromatography was performed using an Isolera One flash purification system (Biotage, Uppsala, Sweden). Pre-packed cartridges, a SNAP HP-Sil (340 g, 100 g, Biotage), a SNAP Ultra (100 g, 25 g, Biotage), and a SNAP KP-C18-HS (400 g, 120 g, Biotage), were used for flash chromatography. Dry load cartridges (100 g, 25 g, 10 g scales, Biotage) manually packed with Sephadex LH-20 and Cosmosil 140C_18_-OPN resins were also used for flash chromatography.

### 3.2. Plant Material

The dried roots of *Asiasarum heterotropoides* were purchased from Kwangmyungdang Medicinal Herbs Co. (Ulsan, Republic of Korea) and identified by Dr. Go Ya Choi, Herbal Medicine Resources Group, Herbal Medicine Research Division, Korea Institute of Oriental Medicine, Republic of Korea. A voucher specimen (KIOM-CRC-2) was deposited at the Cancer Research Team, KM-Based Herbal Drug Development Group, Herbal Medicine Research Division, Korea Institute of Oriental Medicine, Republic of Korea.

### 3.3. Extraction and Isolation

The plant material (10 kg) was ground and extracted with 80% EtOH (40 L for 48 h, three times) by maceration at room temperature. The extracts were filtered (Whatman filter paper, No. 2, Whatman International, Maidstone, UK), concentrated (20 L scale, 40 °C, EYELA rotary evaporation system, Tokyo Rikakikai, Tokyo, Japan), and dried (WiseVen vacuum oven, WOW-70, Daihan Scientific, Seoul, Republic of Korea) to produce an EtOH extract (769 g), which was suspended in distilled water and subsequently partitioned with organic solvents to generate *n*-hexane- (257 g), EtOAc- (52 g), and water-soluble extracts (459 g). The EtOAc-soluble extract was fractionated by flash chromatography using a SNAP-HP-Sil cartridge (340 g, *n*-hexane–EtOAc, 90:10 to 50:50, CHCl_3_–MeOH, 90:10 to 70:30, v/v) to obtain 31 subfractions (F01-F31). F06 (605.4 mg) was fractionated by a flash chromatography system with a SNAP Ultra cartridge (100 g, *n*-hexane–EtOAc, 80:20, v/v) to obtain 6 subfractions (F0601-0606). Further chromatographic separation of these subfractions was also performed using a flash chromatography system with SNAP Ultra (100 g, *n*-hexane–EtOAc, 80:20, v/v) and Cosmosil 140C_18_-OPN (25 g scale × 2, MeOH–Water, 65:35, v/v) cartridges to give compound **11** (11.3 mg). F11 (800.0 mg) was fractionated by a flash chromatography system using a Cosmosil 140C_18_-OPN cartridge (100 g scale, ACN–Water, 5:95 to 40:60, v/v) to give 15 subfractions (F11-01-F11-15). Subfraction F11-03 (261.0 mg) was separated by a flash chromatography system using Cosmosil 140C_18_-OPN (100 g scale, MeOH–Water, 20:80, v/v) and SNAP Ultra (25 g × 2, CHCl_3_–MeOH–Water, 9:1:0.1, v/v) cartridges to obtain compounds **17** (3.0 mg), **19** (22.1 mg), and **20** (5.0 mg). Compounds **8** (40.0 mg) and **9** (76.2 mg) were recrystallized from F1106 (81.8 mg) and F1109 (43.3 mg), respectively. F12 (1.2 g) was subjected to flash chromatography with a SNAP-KP-C18-HS cartridge (120 g, MeOH–Water, 20:80 to 30:70 to 60:40, v/v) to obtain 9 subfractions (F1201-F1209). Subfraction F1205 (132.9 mg) was separated by a flash chromatography system with a SNAP Ultra cartridge (100 g, CHCl_3_–MeOH–Water, 95:5:0.1, v/v) to afford compound **18** (9.0 mg). Fraction F1208 (83.8 mg) was subjected to flash chromatography using SNAP Ultra (100 g, CHCl_3_–ACN, 98:2 to 90:10, v/v) and Cosmosil 140C_18_-OPN (10 g scale × 3, MeOH–Water, 65:35, v/v) cartridges to give compound **12** (29.0 mg). F13 (1.0 g) was fractionated by a flash chromatography system (Cosmosil 140C_18_-OPN cartridge, 100 g scale, MeOH–Water, 35:65 to 60:40, v/v) to afford 21 subfractions (F1301-F1321). Compound **14** (7.6 mg) was separated from F1312 (25.9 mg) by repeated flash chromatography using SNAP Ultra (25 g × 2, CHCl_3_–ACN, 90:10, v/v) and Sephadex LH-20 (10 g scale × 4, MeOH–Water, 10:90 to 50:50, v/v) cartridges. F16 (828.3 mg) was fractionated by a flash chromatography system using a Cosmosil 140C_18_-OPN cartridge (100 g scale, MeOH–Water, 30:70 to 70:30, v/v) to obtain 11 subfractions (F1601-F1611). Fraction F1606 (29.5 mg) was subjected to flash chromatography with a SNAP Ultra cartridge (25 g × 2, CHCl_3_–ACN, 90:10, v/v) and a Cosmosil 140C_18_-OPN cartridge (10 g scale × 3, MeOH–Water, 10:90 to 70:30, v/v) to give compound **7** (3.0 mg). Compound **13** (3.6 mg) was separated from F1608 (62.6 mg) by repeated flash chromatography using SNAP Ultra (100 g, CHCl_3_–ACN, 19:1, v/v) and Cosmosil 140C_18_-OPN (25 g scale × 2, MeOH–Water, 10:90 to 70:30, v/v) cartridges. Chromatographic separation of F17 (2.0 g) was performed using a flash chromatography system with a SNAP Ultra cartridge (100 g, CHCl_3_–MeOH–Water, 95:5:0.5, v/v) to produce 12 subfractions (F1701-F1712). Compound **10** (8.0 mg) was separated by a flash chromatography system (Cosmosil 140C_18_-OPN cartridge, 100 g scale, MeOH–Water, 30:70 to 100:0, v/v) from F1706 (100.2 mg), and compound **4** (4.0 mg) was purified by precipitation from F1704 (80.0 mg). Flash chromatography of F20 (1.3 g) was carried out using a Cosmosil 140C_18_-OPN cartridge (100 g scale, MeOH–Water, 30:70 to 80:20, v/v) to produce 21 subfractions (F2001-F2021). Further chromatographic separations of F2004 (150.2 mg) and F2010 (80.5 mg) were also performed by flash chromatography using SNAP 100 g Ultra cartridges with solvent mixtures CHCl_3_–ACN (80:20, v/v) and CHCl_3_–MeOH–Water (8:2:0.2, v/v) to give compounds **22** (19.7 mg) and **6** (8.0 mg), respectively. Fraction F2014 (56.6 mg) was subjected to flash chromatography (Cosmosil 140C_18_-OPN cartridge, 100 g scale, MeOH–Water, 75:25, v/v) to obtain 4 subfractions (F201401-F201404). Compound **5** (2.0 mg) was obtained from F201402 (20.2 mg) through precipitation. F23 (9.5 g) was fractionated using a flash chromatography system with a SNAP HP-Sil cartridge (340 g, CH_2_Cl_2_–ACN, 90:10 to 50:50, CH_2_Cl_2_–MeOH, 95:5 to 50:50, v/v) to afford 20 subfractions (F2301-F2320). Chromatographic separation of F2306 (238.3 mg) was carried out by a flash chromatography system using a SNAP Ultra cartridge (100 g, CHCl_3_–MeOH, 97:3 to 95:5, v/v) to give 8 subfractions (F230601-F230608). Further chromatographic separations of these subfractions were also performed by a flash chromatography system using Cosmosil 140C_18_-OPN (25 g scale × 2, MeOH–Water, 10:90 to 40:60, v/v), Sephadex LH-20 (10 g × 4 scales, MeOH–Water, 90:10, v/v), and SNAP Ultra (10 g × 3, CHCl_3_–MeOH–Water, 9:1:0.05, v/v) cartridges to provide compounds **1** (14.9 mg) and **3** (8.1 mg). Fraction F2308 (323.8 mg) was fractionated by a flash chromatography system with a SNAP HP-Sil cartridge (100 g, CHCl_3_–MeOH–Water, 9:1:0.05 to 9:1:0.1, v/v) to obtain 10 subfractions (F230801-F230810). Compound **2** (5.0 mg) was purified from F230805 (90.7 mg) by flash chromatography with SNAP KP-C18-HS (120 g, MeOH–Water, 30:70 to 45:55, v/v) and Cosmosil 140C_18_-OPN (100 g scale, MeOH–Water, 20:80 to 40:60, v/v) cartridges. F27 (2.2 g) was fractionated by a flash chromatography system (Cosmosil 140C_18_-OPN cartridge, 100 g scale, MeOH–Water, 5:95 to 20:80) to give 33 subfractions (F2701-F2733). Fractions F2711 (140.5 mg) and F2719 (130.0 mg) were separated by a flash chromatography system using Cosmosil 140C_18_-OPN cartridges (100 g scale) eluted with solvent systems MeOH–Water (15:85 to 20:80 to 25:75, v/v) and ACN–Water (20:80, v/v) to give compounds **21** (18.2 mg) and **16** (14.2 mg), respectively. Compound **15** (16.0 mg) was obtained by precipitation from F2715 (52.5 mg).

### 3.4. Characterization Data of (7S,8R,7'S,8'S)-3-Methoxy-3',4'-methylenedioxy-7,9'-epoxylignane-4,7',9-triol (**1**)

Amorphous solid. 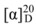 +39.2 (c 0.1, MeOH); UV (MeOH) λ_max_ (log ε) 227 (4.92), 283 (4.44) nm; CD (MeOH, c = 5.3 × 10^−^^3^ M) Δε(nm): +19.9 (245), +8.4 (293); ^1^H-NMR (CDCl_3_, 500 MHz) δ 6.86 (3H, m, overlapping, H-2'/H-5/H-2), 6.79 (1H, m, overlapping, H-6), 6.77 (1H, m, overlapping, H-6'), 6.75 (1H, d, J = 8.0 Hz, H-5'), 5.96 (2H, s, -OCH_2_O-), 5.85 (1H, brs, 4-OH), 4.41 (1H, d, J = 9.5 Hz, H-7'), 4.34 (1H, d, J = 9.0 Hz, H-7), 3.89 (3H, s, OMe), 3.69 (1H, m, H-9a), 3.60 (2H, m, H-9') 3.56 (1H, m, H-9b), 2.54 (1H, m, H-8'), 2.25 (1H, m, H-8); ^13^C-NMR (CDCl_3_, 125 MHz) δ 148.0 (C-3'), 147.5 (C-4'), 146.8 (C-3), 145.6 (C-4), 136.8 (C-1'), 132.5 (C-1), 120.3 (C-6'), 119.7 (C-6), 114.3 (C-5), 108.8 (C-2), 108.1 (C-5'), 106.8 (C-2'), 101.2 (-OCH_2_O-), 84.5 (C-7), 76.9 (C-7'), 70.3 (C-9'), 63.1 (C-9), 56.0 (3-OMe), 55.7 (C-8), 53.5 (C-8'); HRESIMS m/z 397.1263 [M+Na]+ (calcd. for C_20_H_22_O_7_Na, 397.1263).

### 3.5. Cell Culture

IMR90 human normal lung fibroblast cells, A549 human cells, E6-1 human Jurkat T lymphoblastic leukemia cells, and Raw264.7 murine macrophage cells were obtained from American Type Culture Collection (ATCC, Manassas, VA, USA). A549 and E6-1 cells were maintained in RPMI1640, and IMR90 and Raw264.7 cells were grown in DMEM. Culture media were supplemented with 10% fetal bovine serum (FBS), 100 units/mL penicillin, and 100 μg/mL streptomycin in 5% (v/v) CO_2_ humidified air at 37 °C. All culture media and supplements were obtained from Invitrogen (Carlsbad, CA, USA).

### 3.6. FOXP3-Lluciferase Stable Cell Line

The FOXP3-luciferase reporter vector (pGL4.17-FOXP3-luc) was a generous gift from Professor Im, S.H. (School of Life Sciences and Immune Synapse Research Center, Gwangju Institute of Science and Technology, Gwangju, Republic of Korea). The conserved noncoding sequences 1 and 3 (CNS1 and CNS3, respectively), which were shown to be critical regulatory elements controlling FOXP3 gene expression [62] were subcloned into the upstream region of luciferase in the pGL4.17 empty vector (Promega, Madison, WI, USA). The pGL4.17-FOXP3-luc vector was transfected into E6-1 cells by electroporation. Drug resistant cells were selected under 800 μg/mL G418 (Invitrogen). Expression of luciferase was induced by stimulation of cells with 5 ng/mL PMA (Sigma, St Louis, MO, USA). The stable and PMA-responsive single clone (FOXP3-Luc#3) was obtained by limited dilution of the parental resistant cells in 96-well culture plates. The FOXP3-Luc#3 clone was maintained at 400 μg/mL G418.

### 3.7. Hypoxia Inducible Factor (HIF)-Luciferase Stable Cell Line

The NIH3T3 cell line (NIH3T3/HIF-luc), which was stably transfected with the HRE-luciferase reporter vector (pHIF1-Luc), was purchased from Panomics (Fremont, CA, USA). pHIF1-Luc was designed to measure the intracellular HIF-1α. Four copies of HRE sequences (5'-GTGACT ACGTGCTGCCTAG-3') were subcloned into the upstream region of luciferase cDNA. Therefore, the binding of HIF-1α proteins to HRE derives the expression of luciferase. NIH3T3/HIF-luc cells were grown in DMEM supplemented with 10% FBS, 100 units/mL penicillin, 100 μg/mL streptomycin, and 100 μg/mL hygromycin B (Invitrogen) in 5% (v/v) CO_2_ humidified air at 37 °C.

### 3.8. Cell Viability

Cell viability was quantified using the Ez-Cytox cell viability assay kit (Daeil Lab Service, Seoul, Republic of Korea) as previously described [63].

### 3.9. NO Assay

Raw264.7 cells were inoculated at 4.5 × 10^5^ cells/well in 48-well tissue culture plates containing 500 μL of fresh medium and grown overnight. The cells were treated with a combination of lipopolysaccharide (LPS, Sigma, 1 μg/mL) and test compound (20 μM). The NO released from LPS-treated cells into the culture medium was quantified using a commercially available NO detection kit (iNtRON Biotechnology, Seoul, Republic of Korea) as described in the manufacturer’s guide.

### 3.10. Luciferase Reporter Assay

After treatment as designated, the cells were transferred to 1.5 mL tubes and pelleted by centrifugation at 500 g for 5 min. Cells were washed twice with ice-cold phosphate-buffered saline (PBS), and WCL was prepared by repeated freezing and thawing in 1X passive lysis buffer (Promega). The insoluble cellular debris was removed by centrifugation at 14,000 rpm for 10 min. Luciferase activities in the WCL were determined using the luciferase assay system (Promega) according to the manufacturer's instructions. Light intensities were monitored using a GloMax luminometer (Promega).

### 3.11. Western Blot Analysis

Changes in intracellular protein levels were examined by Western blotting as previously described [9]. In short, A549 cells were cultured under normoxic (20% O_2_) or hypoxic (1% O_2_) environments, and WCL was prepared using ice-cold RIPA buffer (Thermo Scientific, Rockford, IL, USA). Equal amounts of proteins (20 μg) were separated on SDS-PAGE gels and electro-blotted onto nitrocellulose membranes. The membranes were blocked with a 5% (w/v) skim milk solution in 0.1% (v/v) Tween-20 PBS and probed with primary antibodies at 4 °C overnight. The primary antibodies were captured by horseradish peroxidase-labeled secondary antibodies. Immuno reactive bands were visualized with the SuperSignal West Femto Kit (Thermo Scientific). All primary antibodies except HIF-1α (Bethyl Laboratories, Montgomery, TX, USA) and β-actin (Santa Cruz Biotechnology, Santa Cruz, CA, USA) were obtained from Cell Signaling Technology (Danvers, MA, USA).

## 4. Conclusions

To discover bioactive natural products from medicinal herbs, *Asiasarum heterotropoides* was analyzed and investigated, affording a new tetrahydrofuran lignan, (7*S*,8*R*,7'*S*,8'*S*)-3-methoxy-3',4'-methylenedioxy-7,9'-epoxylignane-4,7',9-triol (**1**), along with 21 known compounds **2**–**22** including two tetrahydrofuran lignans **2** and **3**, two aristolactams **4** and **5**, an aristolochic acid derivative **6**, seven tetrahydrofurofuran lignans **7**–**12**, three flavanones **14**–**16**, two monoterpenes **17** and **18**, and four phenolics **19**–**22** by chromatographic separation techniques. The structures of **1**–**22** were elucidated using their spectroscopic data, including NMR. In this study, we report for the first time the isolation of 14 of these compounds (**1**–**3**, **7**, **10**, **12**–**17**, **19**, **21**, and **22**) from *A. heterotropoides*. All of the compounds **1**–**22** were evaluated for their biological activities using *in vitro* analyses. Remarkably, 7-methoxyaristololactam IV (**5**) and (7*α*,7'*β*,8*α*,8'*α*)-3,4-methylenedioxy-3',4'-dihydroxy-7,9':7',9-diepoxylignane (**7**) inhibited the growth of A549 human NSCLC cells. Furthermore, compounds **5** and **7** both showed significant inhibitory effects on nitric oxide production, FOXP3 promoter activation, and HIF-1α transcriptional activity. This finding could be useful in developing anticancer agents using this medicinal herb and its active compounds.
